# Immune status is prognostic for poor survival in colorectal cancer patients and is associated with tumour hypoxia

**DOI:** 10.1038/s41416-020-0985-5

**Published:** 2020-07-20

**Authors:** Stephanie G. Craig, Matthew P. Humphries, Matthew Alderdice, Victoria Bingham, Susan D. Richman, Maurice B. Loughrey, Helen G. Coleman, Amelie Viratham-Pulsawatdi, Kris McCombe, Graeme I. Murray, Andrew Blake, Enric Domingo, James Robineau, Louise Brown, David Fisher, Matthew T. Seymour, Phil Quirke, Peter Bankhead, Stephen McQuaid, Mark Lawler, Darragh G. McArt, Tim S. Maughan, Jacqueline A. James, Manuel Salto-Tellez

**Affiliations:** 1 Precision Medicine Centre of Excellence, Centre for Cell Research and Cell Biology, Queen’s University Belfast, Belfast, Northern Ireland; 2 Leeds Institute of Medical Research at St James’s, University of Leeds, Leeds, UK; 3 Department of Cellular Pathology, Royal Victoria Hospital, Belfast Health and Social Care Trust, Belfast, Northern Ireland; 4 Centre for Public Health, Queen’s University Belfast, Belfast, Northern Ireland; 5 Pathology, School of Medicine, Medical Sciences and Nutrition, University of Aberdeen, Aberdeen, Scotland; 6 CRUK/MRC Oxford Institute for Radiation Oncology, Oxford University, Oxford, England; 7 MRC Clinical Trials Unit, University College London, London, UK; 8 Division of Pathology, University of Edinburgh, Edinburgh, Scotland

**Keywords:** Cancer microenvironment, Colorectal cancer, Tumour biomarkers

## Abstract

**Background:**

Immunohistochemical quantification of the immune response is prognostic for colorectal cancer (CRC). Here, we evaluate the suitability of alternative immune classifiers on prognosis and assess whether they relate to biological features amenable to targeted therapy.

**Methods:**

Overall survival by immune (CD3, CD4, CD8, CD20 and FOXP3) and immune-checkpoint (ICOS, IDO-1 and PD-L1) biomarkers in independent CRC cohorts was evaluated. Matched mutational and transcriptomic data were interrogated to identify associated biology.

**Results:**

Determination of immune-cold tumours by combined low-density cell counts of CD3, CD4 and CD8 immunohistochemistry constituted the best prognosticator across stage II–IV CRC, particularly in patients with stage IV disease (HR 1.98 [95% CI: 1.47–2.67]). These immune-cold CRCs were associated with tumour hypoxia, confirmed using CAIX immunohistochemistry (*P* = 0.0009), which may mediate disease progression through common biology (*KRAS* mutations, CRIS-B subtype and *SPP1* mRNA overexpression).

**Conclusions:**

Given the significantly poorer survival of immune-cold CRC patients, these data illustrate that assessment of CD4-expressing cells complements low CD3 and CD8 immunohistochemical quantification in the tumour bulk, potentially facilitating immunophenotyping of patient biopsies to predict prognosis. In addition, we found immune-cold CRCs to associate with a difficult-to-treat, poor prognosis hypoxia signature, indicating that these patients may benefit from hypoxia-targeting clinical trials.

## Background

The immune context of the tumour microenvironment is a recognised hallmark of cancer development, recurrence and response to therapy.^1^ The literature in particular denotes the importance of adaptive immunity in cancer-related outcomes, given that upregulation of its inflammatory mediators is consistently associated with improved prognosis.^2,3^

This has been the rationale behind the development of colorectal cancer (CRC)-specific immune-related prognostication systems that aim to establish the likelihood of disease progression.^4–6^ The feasibility of at least one of these algorithms as a diagnostic test within routine clinical practice has been validated in a large multicentre, multi-cohort, retrospective study for stage I–III colon cancer.^5^ However, in spite of its potential clinical deployment, it remains unclear if the success of immune prognostication in CRC, in the absence of receiving immunotherapy, is based on the increased expression of specific immune biomarkers alone, or the cumulative sum of the numerous immune responses in the tumour.^7^

In this study, we describe the assessment of immune (CD3, CD4, CD8, CD20 and FOXP3) and immune-checkpoint (ICOS, IDO-1 and PD-L1) biomarkers using digital image analysis in stage II–IV CRC patients (*n* = 1724). We evaluate which is the most useful biomarker, or a combination thereof, to predict survival in CRC at diagnosis. We identify the biology behind the prognostic groups by analysing their mutational profile and their relationship to the consensus molecular and CRC intrinsic subtypes (CMS and CRIS, respectively). We complement this by conducting differential gene expression analysis, gene set enrichment analysis (GSEA) and orthogonal validation.^8,9^ Using these data, we determine which characteristics of the tumour microenvironment mediate the immune response and ultimately patient outcome.

## Materials and methods

### Patients

The immune component of the tumour microenvironment was assessed in three retrospectively identified cohorts of patients with resections of primary CRC, ranging from stages II to IV (*n* = 1724). Discovery was performed in the population-representative Epi700 CRC cohort that consists of stage II and III CRC patients (*n* = 661) who underwent surgery in Northern Ireland from 2004 to 2008 (NIB13/0069, NIB13/0087, NIB13/0088 and NIB15/0168). The results were cross-validated in the Grampian CRC cohort that consists of stage II–III CRC patients (*n* = 678) diagnosed within the Grampian National Health Service Scotland from 1994 to 2009, accessed through the Grampian Biorepository (TR000157; OREC 17/YH/0415). Study methodology was then applied de novo (in collaboration with the Stratification in Colorectal Cancer (S:CORT) consortium) to the S:CORT FOCUS cohort of stage IV CRC patients (*n* = 385), who were enrolled in the MRC FOCUS clinical trial (OREC 15/EE/0241).

CRC patients in the Epi700 CRC and Grampian CRC cohorts were surgically managed with or without chemotherapy in accordance with contemporaneous treatment guidelines at the time of diagnosis. Details of the MRC FOCUS trial cohort have been reported in detail elsewhere (Supplementary Fig. S1).^10^ Microsatellite-instability (MSI) status was assessed by PCR in the Epi700 CRC cohort, immunohistochemistry in the Grampian CRC cohort using antibodies (MLH1 and MSH2) and next-generation sequencing in the S:CORT FOCUS cohort as described previously.^11,12^ Overall survival (OS) was used as the primary clinical endpoint in all three cohorts. OS was defined as the time from either diagnosis (Epi700 CRC and Grampian CRC cohorts) or randomisation (S:CORT FOCUS cohort) until the time of death. Data were right-censored for patients still alive at the date of the last known follow-up.

### Procedures

Patient material for the three cohorts under assessment was provided as 4-µm, formalin-fixed paraffin-embedded tissue sections from tissue microarrays (TMA) containing 0.6–1.0-mm tumour cores. Immune analysis assays in tissue have been developed primarily for use in full-face tissue sections. However, patient material for the three cohorts under assessment was only available in TMA format with variable TMA design, and only full-face sections from patients within the discovery cohort were available to confirm TMA findings. TMA construction of the Epi700 CRC and Grampian CRC cohorts has been reported previously.^11,12^ In brief, TMAs for the Epi700 CRC cohort were constructed using cores taken from tumour epithelial-rich areas (central tumour) and the invasive margin, whereas TMA cores representing the invasive margin were not available for either the Grampian CRC or S:CORT FOCUS cohorts. TMAs for the S:CORT FOCUS cohort were constructed using 0.6-mm cores taken in triplicate from epithelial-rich tumour regions in formalin-fixed paraffin- embedded blocks for each patient using a Beecher manual arrayer (Beecher Instruments Inc., Sun Prairie, Wisconsin, USA).

All work on the TMA sections was undertaken blinded to clinical outcomes in the Precision Medicine Centre of Excellence at Queen’s University Belfast using standardised operating procedures for immunohistochemical staining, digital slide scanning and digital image analysis to reduce potential sources of bias in data collection. All procedures were reviewed and agreed by senior consultant pathologists (J.J., M.B.L. and M.S.T.).

Immunohistochemistry was performed for adaptive immune (CD3, CD4, CD8, CD20 and FOXP3) and immune-checkpoint (ICOS, IDO-1 and PD-L1) biomarkers on either the Ventana BenchMark XT (Ventana Medical Systems, Oro Valley, Arizona, USA) or Leica BOND-MAX (Leica Biosystems, Wetzlar, Germany) automated immunostainers. Multiplex immunofluorescence for CD3, CD4 and CD8 was conducted using opal chemistry on the Leica BOND-MAX (Leica Biosystems, Wetzlar, Germany) automated immunostainer. Antibody optimisation was conferred and agreed upon with senior consultant pathologists (J.J. and M.S.T.) prior to the study (Supplementary Table S1). All immunostained slides were scanned using a Leica Aperio AT2 at ×40 magnification or an Akoya Vectra Polaris at ×20 magnification using MOTIF scanning protocols if immunoflorescently stained. All scans were independently reviewed for quality and consistency by trained senior technicians (V.B. and A.V.P.) and a consultant clinical scientist (SMcQ), before they were considered for digital image analysis. Immunohistochemistry for the hypoxia biomarker CAIX on the S:CORT FOCUS TMAs was carried out by the Leeds Institute of Medical Research at St James’s using the DAKO Autostainer Link 48 (Agilent Technologies, Santa Clara, California, USA) (Supplementary Table S1).

**Fig. 1:**
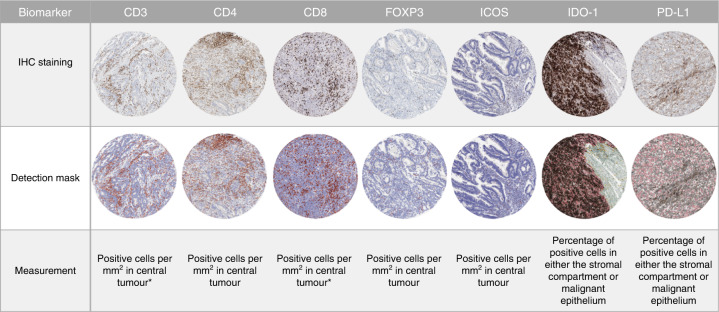
Representative images of immune and immune-checkpoint biomarker staining, and their cell detection mask overlays used in their digital image analysis. Methods of assessment are as listed. *CD3- and CD8-expressing cells as a value of positive cells per mm^2^ were also assessed within the invasive margin when appropriate.

Assessment of all biomarkers was undertaken using open-source software QuPath version 0.2.0.m6 (Fig. 1).^13^ Full-face sections cut at 4 µm were annotated with the assistance of a senior consultant pathologist (M.S.T.). Assessment of the invasive margin on full-face CRC tissue sections was defined as a 500-µm border, taken from the outermost edge of the malignant glands.^14^ Quantification of CD4-expressing cells in singleplex immunohistochemistry and multiplex immunofluorescence experiments by digital image analysis was compared with “calculated” CD4 cell counts, obtained from subtracting the number of CD8- from CD3-expressing cells, using normal colonic epithelium. Multiplex findings were validated on full-face resection specimens of CRC tissue. Assessment of the immune biomarkers was taken as an average over the number of cores available. All analysed images were independently reviewed for quality-control purposes, with at least 20% being reviewed by a pathologist prior to data export. For discovery purposes, all biomarkers assessed were dichotomised using Youden’s J statistic by ROC curve analysis based on survival outcomes.

CRC data matrices for gene expression analysis in the S:CORT FOCUS cohort were provided by the S:CORT consortium. In brief, mutational data were generated for common driver genes *APC, BRAF, KRAS, NRAS, PIK3CA* and *TP53* using next-generation sequencing (at Wellcome Trust Sanger Institute), while gene expression data were generated using the Almac Xcel array (at Almac Diagnostics, Craigavon).^15^ Gene expression data were pre-processed and normalised using robust multi-array analysis and probes collapsed by mean. CRIS and CMS classification was implemented using R packages CRISclassifier and CMSclassifier, respectively.^8,9^ Differential gene expression analysis, principal component analysis (PCA) and hierarchical clustering were performed using Partek® Genomics Suite® software version 6.6. A list of differentially expressed probes was generated using analysis of variance (ANOVA) with a threshold of +/−1.5-fold change and an adjusted p value of less than 0.05. Heatmaps with hierarchical clustering were constructed using Ward’s linkage and Euclidean distance. Row-expression values were standardised to a mean of zero and scaled to a standard deviation of one. GSEA using the WINTERS_HYPOXIA metagene signature was performed using the Broad Institute software (http://software.broadinstitute.org/gsea/index.jsp).

### Statistical analysis

All statistical analyses were conducted using R version 3.5.1. The missing indicator method was used to handle missing clinical data.^16^ Assessment for correlation, accuracy and precision of sample classification without the invasive margin was conducted. An overall 5-year survival analysis was visualised using the Kaplan–Meier method with log-rank *P* values. Cox proportional-hazard models were used to calculate hazard ratios (HR) and associated 95% confidence intervals (CI) for univariate and multivariable analysis for potential confounders as conducted elsewhere (age, sex, stage, MSI status and treatment).^5^ Competitive model selection on non-nested immune biomarker models was based on log likelihood (LL) using second-order Akaike’s Information Criterion (AICc). Models with a difference in AIC of four or more were not considered competitive. ANOVA and Pearson’s chi-squared were used to test for differences in continuous and categorical variables, respectively, across the immune subgroups. Pearson’s product–moment and Spearman rank-order correlations were conducted to assess linear and monotonic relationships, respectively, between biomarkers assessed.

The reporting standards of this study fulfil recommendations set by the STROBE statement for reporting of observational studies and the REMARK guidelines for tumour prognostic studies.^17,18^

## Results

### Determination of collinearity for biomarker combinations

**Fig. 2:**
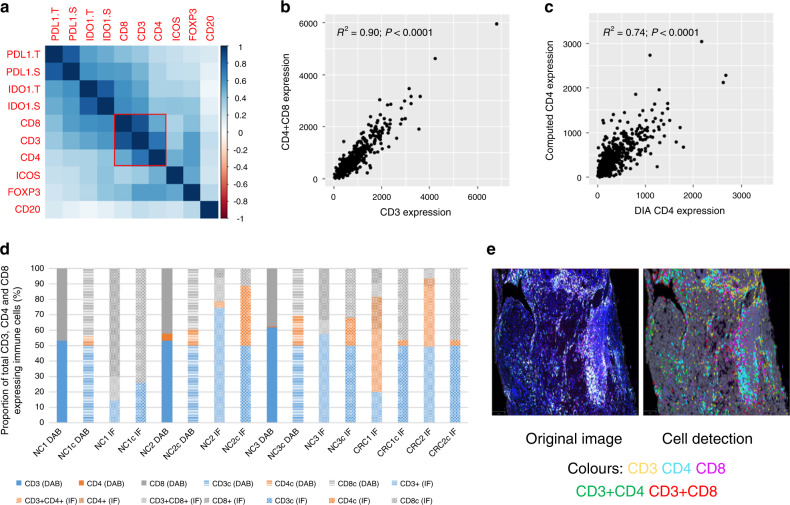
Evaluation of immune and immune checkpoint biomarker collinearity in colorectal cancer. Correlation matrix of the immune and immune checkpoint biomarkers assessed in the Epi700 discovery cohort (**a**). Plot demonstrating the relationship between CD3 and a combination of CD4- and CD8-expressing cells when CD4 and CD8 expression is added together (**b**). Plot demonstrating the relationship between CD4 generated by digital image analysis (DIA) and a computed CD4 score by subtracting CD8 from CD3 (**c**). Stacked bar graph demonstrating the relative difference in CD4 quantification when assessed directly using either immunohistochemistry or multiplex immunofluorescence to quantify CD3-, CD4- and CD8-expressing cells compared with a computed CD4 score obtained via the subtraction of CD8 from CD3 cell counts in the same sample (**d**). Representative image of CRC tissue stained for CD3, CD4 and CD8 using multiplex immunofluorescence, with and without cell detection mask overlay, demonstrating weakly positive cells that would be classified as expressing CD3 only, CD4 only or CD8 only by digital image analysis, as well as the expected dual-positive CD3 and CD4, or CD3 and CD8 phenotypes (**e**). Pearson’s product–moment correlation was used to compare linear relationships between immune biomarkers in **a**. The corresponding correlation matrix was ordered according to the angular order of the eigenvectors; outlined in red are variables with a strong, significant correlation (*R*^2^ > 0.7, *P* < 0.05). Spearman rank-order correlation was used to assess monotonic relationships between variables of interest in **b** and (**c**). IDs for samples compared in the stacked bar graph* (**d**): NC1 = normal colonic epithelium case 1; NC2 = normal colonic epithelium case 2; NC3 = normal colonic epithelium case 3; CRC1 = colorectal cancer case 1; CRC2 = colorectal cancer case 2; DAB = singleplex immunohistochemical DAB staining assessment; IF = multiplex immunofluorescent staining assessment. *If the case ID is followed by small “c”, e.g., NC1c, then the proportion of CD4-expressing cells expected for that case has been calculated by subtracting the cell count for CD8- from CD3-expressing cells in the sample.

CD3-, CD4-, CD8-, CD20-, FOXP3-, ICOS-, IDO-1- (tumour and stroma) and PD-L1- (tumour and stroma) expressing cells were evaluated using singleplex IHC and quantified for use in survival analysis as described in Fig. 1. Many of these biomarkers are known to be simultaneously upregulated; therefore, collinearity was used to indicate a cumulative immune response (Fig. 2a). Indeed, all biomarkers demonstrated some degree of positive correlation with each other. Of the biomarkers assessed, the most significant relationships were observed between CD4 and CD8 IHC to CD3 IHC (*R*^2^ = 0.87, *P* < 0.0001 for CD8 IHC to CD3 IHC and *R*^2^ = 0.73, *P* < 0.0001 for CD4 IHC to CD3 IHC). Quantification of IDO-1 IHC in the stroma was also found to demonstrate a moderate correlation with CD8 IHC (*R*^2^ = 0.62, *P* < 0.0001).

The most successful immune-based prognostication algorithms often combine the assessment of CD3 and CD8 IHC to predict survival.^5^ Total CD4- and CD8-expressing cells are analogous for cumulative CD3 expression; therefore, we anticipate that many biomarker studies may in fact confer CD4 expression through quantitation of CD3 and CD8 IHC alone.^19^ We confirmed, using IHC in the Epi700 cohort, that the sum of total CD4- and CD8-positive cells per mm^2^ has a strong positive relationship with the number of CD3-positive cells per mm^2^ (*R*^2^ = 0.90, *P* < 0.0001, Fig. 2b). Surrogate CD4 expression, calculated by the subtraction of CD8 from CD3 IHC, was also found to be representative of CD4 IHC results; however, the relationship was diminished in comparison (*R*^2^ = 0.74, *P* < 0.0001, Fig. 2c). To understand this better, we assessed the relationship between CD3-, CD4- and CD8-expressing cells in a separate cohort of normal colonic epithelium and colorectal cancer by singleplex IHC and multiplex immunofluorescence. Both techniques showed that calculation of a surrogate CD4 result from CD3 and CD8 cell counts was prone to misestimation of CD4-expressing cells (Fig. 2d). Multiplex immunofluorescence demonstrated that this difference arose due to limitations in the detection chemistry for accurately quantifying weakly positive CD4- and CD8-expressing cells present in the sample (Fig. 2e).

### Determination of an immune-cold phenotype that is predictive of poor overall survival

To facilitate survival analysis, all biomarkers assessed were dichotomised using ROC curve analysis within the Epi700 cohort. Univariate and multivariable Cox proportional hazards for these biomarkers were carried out for each of the individual biomarkers prior to testing combinations. All the biomarkers assessed were individually predictive of survival by themselves, and when adjusted for age, sex, MSI status, stage and treatment, except PD-L1 stromal IHC (Supplementary Table S2). In addition, based on the results for their individual survival analysis and collinearity of the biomarkers assessed, combinations of dichotomised densities for CD3 and CD8 IHC, CD3, CD4 and CD8 IHC and CD3, CD4, CD8 and IDO-1 stromal IHC were assessed for survival outcomes (Supplementary Table S2). Of these analyses, assessment of low-density cell counts for CD3, CD4 and CD8 IHC was found to produce the greatest risk of mortality (HR 1.70, 95% CI: 1.28–2.27).

Due to the variability in TMA design across cohorts, quantification of CD3 and CD8 IHC was assessed in a subset of patients (*n* = 20) in the Epi700 cohort on full-face sections and in TMAs created from the same block (Supplementary Fig. S2A). Moderate–strong correlations were found for both CD3 and CD8 IHC quantified in the invasive margin and central tumour (CD3 invasive margin *R*^2^ = 0.65, *P* < 0.0001; CD3 central tumour *R*^2^ = 0.94, *P* < 0.0001; CD8 invasive margin *R*^2^ = 0.66, *P* < 0.0001; CD8 central tumour *R*^2^ = 0.97, *P* < 0.0001; Supplementary Fig. S2B–E). Based on these data, use of TMA cores did not appear to influence assessment of CD3 or CD8 IHC, thereby, validating the use of TMAs for the current study. To consider if assessment of CD3 and CD8 IHC was warranted in both the invasive margin and central tumour, a comparison of the percentile rank (i.e., the combined density of CD3 and CD8 IHC per patient ranked by order of increasing expression, with and without the inclusion of results from the invasive margin) was conducted. A strong correlation in the percentile rank of these biomarkers in both areas was observed; therefore, a score based on central tumour expression alone following the immunohistochemical quantification of CD3- and CD8-expressing cell density was considered sufficient for use in the other sample cohorts (Grampian CRC and S:CORT FOCUS) to classify patients (*R*^2^ = 0.89, *P* < 0.0001; Supplementary Fig. S2F). In order to create an immune classifier that could be replicated for single-sample analysis, the accuracy and precision of patient classification using biomarker density for stratification were compared with patient stratification using percentiles for CD3 and CD8 IHC. Patient stratification defined by density, using optimised cut-offs (thresholds of 300 and 350 positive cells per mm^2^ for CD3 and CD8, respectively), had an accuracy of 88.47% (95% CI: 85.51–91.01) and precision of 93.99% (95% CI: 91.90–95.56) for predicting the low CD3 and CD8 IHC percentile group (percentile cut-off of 25%). Low-density CD3 and CD8 IHC tumours were then further stratified by the addition of CD4 IHC (threshold of 100 positive cells per mm^2^).

**Fig. 3:**
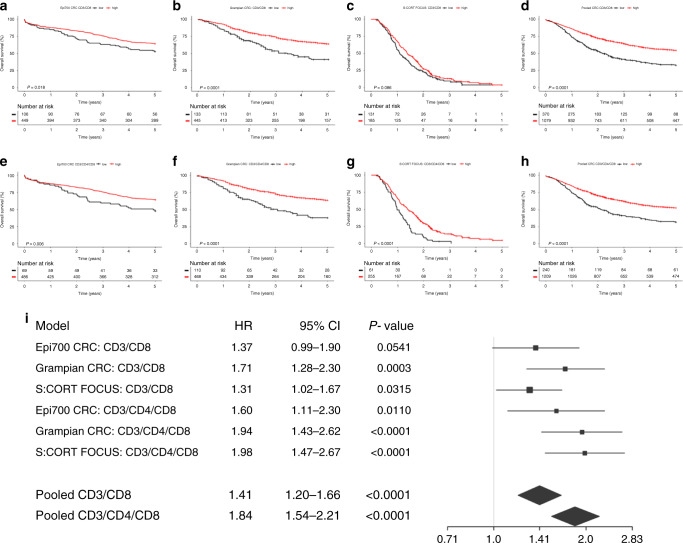
Survival estimates in the study cohorts assessed using either CD3 and CD8 immunohistochemistry (IHC) or CD3, CD4 and CD8 IHC. Kaplan–Meier plots demonstrating univariate survival for immune subgroups defined by assessment of either CD3 and CD8 IHC or CD3, CD4 and CD8 IHC (**a**–**h**). Forest plot showing adjusted hazard ratios (95% CI) and corresponding *P* values for multivariable analysis of immune subgroups defined by assessment of either CD3 and CD8 IHC or CD3, CD4 and CD8 IHC; multivariable analysis was adjusted for age, sex, MSI status, stage and treatment in each cohort (**i**). The pooled analyses for immune subgroups defined by assessment of either CD3 and CD8 IHC or CD3, CD4 and CD8 IHC are stratified by cohort in the multivariable model. Differences in Kaplan–Meier survival curves are presented as log-rank *P* value. Expression cut-offs were optimised in the Epi700 CRC cohort and applied throughout; CD3 = 300, CD4 = 100, CD8 = 350 positive cells per mm^2^. Only patients with combined low expression for either CD3 and CD8 IHC or CD3, CD4 and CD8 IHC were considered to have low expression of these biomarkers.

As nearly all the biomarkers and their combinations were significant for predicting overall survival, competitive model selection was utilised. Using AICc on dichotomised immune biomarker subgroups determined that stratification by combinations of CD3, CD4 and CD8 IHC was the most competitive model for patient stratification (Delta AICc <4). Combinations of CD3, CD4 and CD8 IHC for predicting outcome were then assessed in the Grampian Cohort and replicated in the S:CORT FOCUS cohort using the fixed threshold (Supplementary Table S3). In all three cohorts, patient stratification combining low-density cell counts for CD3, CD4 and CD8 IHC was consistently found to be one of the most competitive models for patient stratification by AICc. In total, we assessed 1,724 surgically resected primary CRC patients ranging from stages II to IV, of which, 1449 (84.05%) had complete results for the whole set of immune biomarkers assessed. Median survival was 5.80 years, 4.33 years and 1.28 years, for the Epi700 CRC, Grampian CRC and S:CORT FOCUS cohorts, respectively. Composition of baseline patient characteristics significantly differed between the three cohorts assessed in the current study; however, the age and gender were similar between the Epi700 and Grampian CRC cohorts, and we expected the S:CORT FOCUS cohort to be significantly different as these patients were enrolled in a clinical trial (Table 1). The baseline characteristics, survival outcomes and immune classifications of the S:CORT FOCUS cohort were well balanced and were representative of the full trial population associated with first-line 5-Fluorouracil (5FU) or Oxaliplatin/5-Fluorouracil (FOLFOX) chemotherapy in MRC FOCUS (Supplementary Table S4). Low-density cell counts for both immune classifiers (CD3 and CD8 IHC and CD3, CD4 and CD8 IHC) were found to be independent predictors of survival when adjusted for covariates in the cohorts assessed and in a pooled analysis stratified by cohort (Fig. 3; Supplementary Table S5). Stratification by low-density cell counts of CD3, CD4 and CD8 IHC demonstrated the most significantly reduced 2-year survival in the S:CORT FOCUS cohort, with only 8.61% of patients alive at 2 years compared with 23.00% of patients when stratified by low-density cell counts of CD3 and CD8 IHC alone (Fig. 3g).

**Table 1:** Baseline characteristics of study patients with immune results, according to cohort.

	Epi700 CRC	Grampian CRC	*P* value (A)	S:CORT FOCUS	*P* value (B)	Pooled CRC
	(*n* = 555)	(*n* = 578)		(*n* = 316)		(*n* = 1449)
Median age (interquartile range)	72 (64–78)	71 (62–78)	0.2938	64 (59–70)	<0.0001	70 (61–77)
*Age*	–	–	0.9010	–	<0.0001	–
<70	238 (42.88%)	251 (43.43%)	–	234 (74.05%)	–	723 (49.90%)
70+	317 (57.12%)	327 (56.57%)	–	82 (25.95%)	–	726 (50.10%)
*Sex*	–	–	0.3606	–	0.0023	–
Male	306 (55.14%)	302 (52.25%)	–	203 (64.24%)	–	811 (55.97%)
Female	249 (44.86%)	276 (47.75%)	–	113 (35.76%)	–	638 (44.03%)
*Stage*	–	–	0.0001	–	<0.0001	–
II	338 (60.90%)	285 (49.31%)	–	0 (0%)	–	623 (43.00%)
III	217 (39.10%)	293 (50.69%)	–	0 (0%)	–	510 (35.20%)
IV	0 (0.00%)	0 (0.00%)	–	316 (100%)	–	316 (21.81%)
*MSI*	–	–	<0.0001	–	<0.0001	–
Stable	392 (70.63%)	473 (81.83%)	–	273 (86.39%)	–	1138 (78.54%)
High	118 (21.26%)	97 (16.78%)	–	12 (3.80%)	–	227 (15.67%)
Missing	45 (8.11%)	8 (1.38%)	–	31 (9.81%)	–	84 (5.80%)
*Adjuvant chemotherapy*	–	–	<0.0001	–	<0.0001	–
No	401 (72.25%)	0 (0.00%)	–	0 (0.00%)	–	401 (27.67%)
Yes	154 (27.75%)	0 (0.00%)	–	316 (100.00%)	–	470 (32.44%)
Missing	0 (0.00%)	578 (100.00%)	–	0 (0.00%)	–	578 (39.89%)

The data are presented as the number of patients (%). Differences in patient characteristics between the study cohorts for the stage-matched Epi700 and Grampian CRC in *P* value (A) and for all the cohorts in *P* value (B) using ANOVA and Pearson’s chi-squared test for continuous and categorical variables, respectively.

### Orthogonal characterisation of a CD3, CD4 and CD8 immune-cold CRC phenotype

In order to assess whether our immune-cold patients were not already defined by a broader molecular signature, we used available CRC tumour mutational and transcription profiles in the S:CORT FOCUS cohort (*n* = 293). We found that patients who were immune-cold were more likely to be *KRAS* mutant (68.97%), have a CRIS-B transcriptional profile (27.59%) and were not associated with either MSI status or CMS subtype (*P* < 0.0179, Table 2). Differential gene expression analysis was performed using ANOVA (*n* = 298). We observed 20 probes to be differentially expressed between immune-cold and immune–not otherwise (NOS) specified patients (adjusted *P* < 0.05). The most common gene detected in the 20-probe list was *SPP1*, occurring four times (ADXEC.2281.C1_x_at, ADXOCEC.14560.C1_at, ADXECAD.5047_at and ADXEC.2281.C2_x_at) out of a possible seven annotated probes (Supplementary Tables S6 and S7). Expression of *SPP1* mRNA was significantly increased in immune-cold patients compared with immune NOS (*P* < 0.0001; Supplementary Fig. S3A).

**Table 2:** MSI status, mutational status and transcriptional subtype of S:CORT FOCUS study patients, according to immune subgroups (immune cold vs. immune NOS).

Variable	Immune cold	Immune NOS	*P* value
	(*n* = 58)	(*n* = 235)	
*MSI status*	–	–	0.5172
Stable	57 (98.28%)	224 (95.32%)	–
High	1 (1.72%)	11 (4.68%)	–
*APC*	–	–	0.6434
Wild type	12 (20.69%)	40 (17.02%)	–
Mutant	46 (79.31%)	195 (82.98%)	–
*BRAF*	–	–	0.6696
Wild type	49 (84.48%)	206 (87.66%)	–
Mutant	9 (15.52%)	29 (12.34%)	–
*KRAS*	–	–	0.0013
Wild type	18 (31.03%)	131 (55.74%)	–
Mutant	40 (68.97%)	104 (44.26%)	–
*NRAS*	–	–	0.3284
Wild type	57 (98.28%)	221 (94.04%)	–
Mutant	1 (1.72%)	14 (5.96%)	–
*PIK3CA*	–	–	0.1391
Wild type	40 (68.97%)	186 (79.15%)	–
Mutant	18 (31.03%)	49 (20.85%)	–
*TP53*	–	–	1.0000
Wild type	16 (27.59%)	63 (26.81%)	–
Mutant	42 (72.41%)	172 (73.19%)	–
*CRIS classification*	–	–	0.0179
CRIS-A	12 (20.69%)	40 (17.02%)	–
CRIS-B	16 (27.59%)	28 (11.91%)	–
CRIS-C	10 (17.24%)	64 (27.23%)	–
CRIS-D	4 (6.9%)	24 (10.21%)	–
CRIS-E	11 (18.97%)	35 (14.89%)	–
Unclassified	5 (8.62%)	44 (18.72%)	–
*CMS classification*	–	–	0.5616
CMS1	6 (10.34%)	25 (10.64%)	–
CMS2	9 (15.52%)	60 (25.53%)	–
CMS3	7 (12.07%)	21 (8.94%)	–
CMS4	18 (31.03%)	61 (25.96%)	–
Unclassified	18 (31.03%)	68 (28.94%)	–

The data are presented as number of patients (%). Differences compared with the immune subgroups using Pearson’s chi-squared test for categorical variables. Immune cold = patient stratification by collective low-density cell counts for CD3, CD4 and CD8 IHC; immune–not otherwise specified (NOS) = any other combination of CD3, CD4 and CD8 IHC expression.

**Fig. 4:**
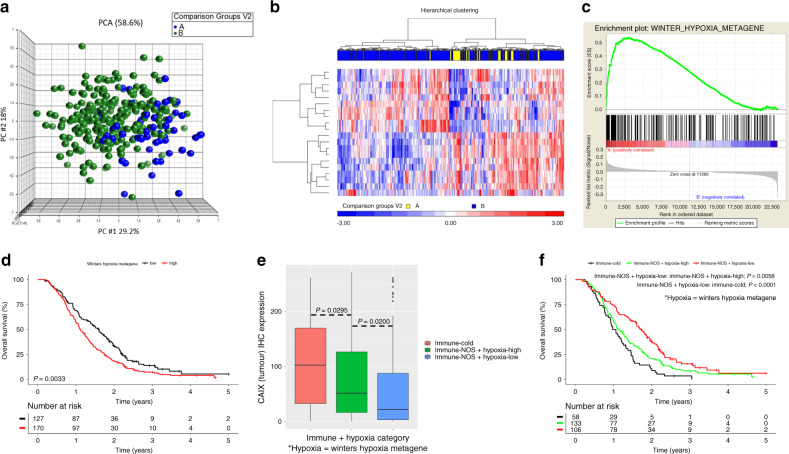
Orthogonal characterisation of a CD3, CD4 and CD8 immune-cold CRC phenotype. PCA (**a**) and heatmap (**b**) demonstrating the distribution and clustering of the 20 most variable probes identified by differential gene expression analysis between immune-cold (Group A) and immune-NOS (Group B) subgroups in the S:CORT FOCUS cohort. GSEA enrichment plot (**c**) for the WINTER_HYPOXIA_METAGENE signature in the immune subgroups. Kaplan–Meier curve of dichotomised Winters Hypoxia Metagene Signature (**d**). Boxplot to demonstrate the relationship between immune subgroups and tumour hypoxia using CAIX IHC expression from the tumour epithelium (**e**). Kaplan–Meier curve of combined immune subgroups and tumour hypoxia (**f**). Differences in immune subgroups were compared using ANOVA. Differences in survival curves are presented as log-rank *P* value. Immune cold = patient stratification by collective low-density cell counts for CD3, CD4 and CD8 IHC; immune–not otherwise specified (NOS) = any other combination of CD3, CD4 and CD8 IHC expression; in **e**, **f** this was further stratified by hypoxia-low = low tumour hypoxia by the Winters Hypoxia Metagene Signature or hypoxia-high = high tumour hypoxia using the Winters Hypoxia Metagene Signature.

PCA and hierarchical clustering of patients using these 20 probes demonstrated separation and clustering of transcriptional profiles for our immune-related groupings (Fig. 4a, b). Many of the differentially expressed genes are known to be associated with hypoxia, including *SPP1*. GSEA for a hypoxia signature (WINTERS_HYPOXIA_METAGENE) found significant upregulation of hypoxia-associated genes within immune-cold patients, confirming this association (adjusted *P* = 0.0005, Fig. 4c). Dichotomised expression of the Winters Hypoxia Metagene signature was significant for predicting overall survival in the S:CORT FOCUS cohort (HR 1.45 [95% CI: 1.13–1.86], *P* = 0.0033). The transcriptomic analysis was orthogonally validated by assessment of CAIX IHC, a robust inducible biomarker for tumour hypoxia, which has been well characterised and is part of the hypoxia signature assessed. CAIX IHC was quantified as the number of positive cells per mm^2^ (*n* = 314). A significant increase in CAIX IHC expression was observed in immune-cold patients compared with immune NOS, thereby confirming the presence of increased tumour hypoxia in these patients (*P* value = 0.0009; Supplementary Fig. S3B-C). While expression of CAIX IHC was not significant for predicting overall survival, the overall trend remained the same (HR 1.27 [95% CI: 0.98–1.65], *P* = 0.0713; Supplementary Fig. S3D). Based on hierarchical clustering observed in Fig. 4b, a subgroup of patients who were both immune NOS and hypoxic were identified following further stratification of immune-NOS tumours by tissue hypoxia (Fig. 4e). Interestingly, these patients were less significantly hypoxic than immune-cold patients (*P* = 0.0295). In view of this observation, we considered these patient groups (immune cold and immune NOS stratified by either high or low hypoxia) for survival analysis in the S:CORT FOCUS cohort (Fig. 4f). A 1.5-fold (HR 1.48 [95% CI: 1.12–1.95], *P* = 0.0058) and 2.2-fold (HR 2.19 [95% CI: 1.55–3.08], *P* < 0.0001) increase in risk of death was found in patients who were immune-NOS hypoxia-high or immune-cold compared with immune-NOS hypoxia-low patients. In this context, the combined assessment of CD3, CD4 and CD8 IHC and tumour hypoxia by the Winters Metagene signature was found to be an independent predictor for survival when adjusted for clinical covariates, *KRAS* status and CRIS-B subtype (Supplementary Table S8).

## Discussion

Spatiotemporal analyses of the immune response have previously highlighted global depression of the T-cell response in the central tumour and invasive margin as stage increases, whilst inversely B-cell expression increases.^2^ Our study found expression of T-helper (CD4) to correlate strongly with CD3- and CD8-expressing cells quantified in the central tumour, when the dichotomised density of CD4-expressing cells was combined with dichotomised densities of CD3- and CD8- expressing cells. We found patient stratification for immune-cold tumours to significantly improve prognostic power for OS across all stages over assessment by dichotomised densities of CD3- and CD8-expressing cells alone. Of the biomarkers assessed, CD3, CD4 and CD8 are principal T-cell lineage commitment biomarkers essential for the adaptive immune response.^19^ Using all three biomarkers provides a more accurate interpretation of the patient’s native adaptive immune response than immunohistochemical assessment of CD3- and CD8-expressing cells alone, and appears to delineate patients who are truly evading the host immune response. We demonstrate using singleplex immunohistochemistry and multiplex immunofluorescence that the correlation of CD4 direct quantification versus CD4 as a post calculation from CD3 and CD8 expression was strong but not perfect, and arises from limitations in the detection chemistry and its quantification using digital image analysis. We found that the specific analysis of the density of CD4-expressing cells in combination with densities of CD3- and CD8-expressing was found to be the best means to stratify patients across three independent cohorts. This illustrates the important but imperfect nature of immunohistochemistry; indeed, the exact analysis of CD4 obviously provides a significant clinical advantage.

In contrast to previous studies, our study focused on the central tumour expression of immune and immune-checkpoint biomarkers only. It was recently demonstrated that assessment of the immune infiltrate for CD3 and CD8 IHC at either the invasive margin or central tumour is predictive of survival.^20^ Indeed, our study confirms that a comparable patient immune classification can be derived without assessment of the invasive margin. This has implications for extrapolation of our immune-cold status as defined by the combined low-density cell counts for CD3, CD4 and CD8 IHC into scoring of endoscopic diagnostic biopsy samples that are taken from the luminal aspect and not the advancing tumour edge.

The robust process exercised in this study to score cancer immune markers allows a clear-cut determination of the clinical relevance of not only our immune-cold subgroup, but also its biological nature. A number of gene expression profiling approaches, including CMS and CRIS, have been successfully applied to define particular CRC molecular subtypes.^8,9^ CRIS classifications offer a more robust measure of describing CRC molecular subtypes compared with CMS, as CRIS transcriptional profiles were developed independent of the tumour stromal content, which can be subject to sampling bias in CMS classifications.^21,22^ We demonstrate for the first time that patients who are immune-cold (when defined by low-density CD3, CD4 and CD8 IHC expression) are most likely to associate with the poor prognosis of CRIS-B transcriptional profile, whilst no association with any particular CMS profile was identified. Patients with CRIS-B tumours are associated with aggressive disease and TGF-β signalling. TGF-β signalling has been previously shown to be enhanced by long-term hypoxia in tissues, and has also been implicated in immune evasion.^23^

This study indicates a significant relationship between tumour hypoxia, the immune response and their combined effect on patient prognosis in CRC, complementing mechanistic studies wherein tumour hypoxia has been shown to mediate immunosuppression.^24,25^ Differential gene expression analysis of the immune-cold subgroup showed *SPP1* mRNA upregulation in immune-cold CRC tumours. *SPP1* mRNA encodes the Osteopontin protein that is an established mediator of tumorigenesis, disease progression and recurrence in cancer, and its expression is known to be influenced by hypoxia, which has been linked previously with immune exclusion.^26–29^ In addition, we determined that our immune-cold patients are more likely to have *KRAS* mutations that have been associated with TGF-β signalling, and therefore hypoxia, through crosstalk of *RAS*, and have previously been demonstrated to have poor prognosis in patients enrolled in the MRC FOCUS trial.^30,31^ Clinical trials are currently underway to assess the clinical applicability of Osteopontin as a blood-based biomarker for prognosis and monitoring response to treatment.^32^ Furthermore, it has been proposed that patients with a hypoxia signature may benefit from more aggressive treatment or improved oxygen levels in tumours.^33^ We speculate that joint assessment of immune-cold status by CD3, CD4 and CD8 IHC in the resection specimen and Osteopontin expression levels in the blood would provide a robust approach to identify hypoxic tumours and monitor in real time their response to therapy.^34^

To conclude, immunohistochemical assessment of CD3- and CD8-expressing cells has set the precedent for clinical assessment of the immune contexture in patients with stage I–III colon cancer. Critically, we demonstrate that assessment for low-density cell counts by CD3 and CD8 IHC should be complemented with CD4 IHC expression analysis to enhance patient prognostication and potential future treatment selection. We also demonstrate that specific analysis of the invasive front may not be necessary, opening the test to easier delivery. In contrast to other proposed methods, we find that addition of CD4 IHC consistently identifies patients with the worst outcomes in stage II–IV CRC. We establish for the first time that immune-cold patients by assessment of CD3, CD4 and CD8 IHC are linked with difficult-to-treat, poor prognosis hypoxic biology, which may be potentially amenable to targeted therapy or monitoring for disease progression.

## Supplementary information

Supplementary Data

## Data Availability

The data are held within the Northern Ireland Biobank and the stratified medicine consortium in colorectal cancer (S:CORT), respectively, and are available on application.
