# Supplementary material for: Immune status is prognostic for poor survival in colorectal cancer patients and is associated with tumour hypoxia
Source: Br J Cancer. 2020 Jul 20;123(8):1280–8. doi: 10.1038/s41416-020-0985-5 (PMC7555485; doi:10.1038/s41416-020-0985-5)
Supplement: Supplementary file 1 — Supplementary Data [file 41416_2020_985_MOESM1_ESM.pdf]

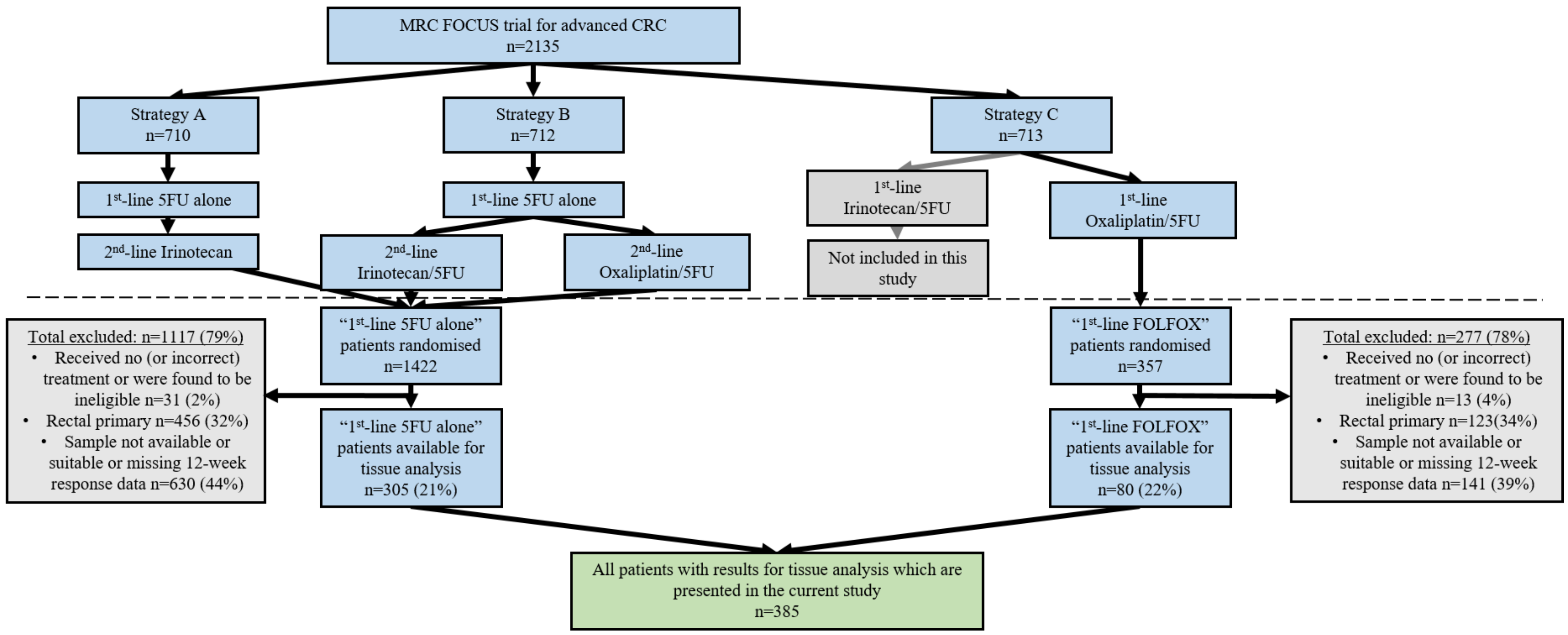

Supplementary Figure S1: MRC FOCUS clinical trial CONSORT diagram.

Patients with advanced CRC were randomly assigned to three treatment strategies—A, B, and C—in equal numbers. Patients assigned to strategies B and C were further randomly assigned to combination chemotherapy that included either Irinotecan or Oxaliplatin. This resulted in a five-arm randomized trial at a ratio of 2:1:1:1:1. Reference 10 provides full details including treatment regimens. Patients assigned to strategies A, B and C who received either first-line 5-Fluorouracil (5FU) or Oxaliplatin/5-Fluorouracil (FOLFOX) were randomised for inclusion in the current study (n=1779), of which a subset (n=385) were selected for tissue/gene expression profiling analysis.

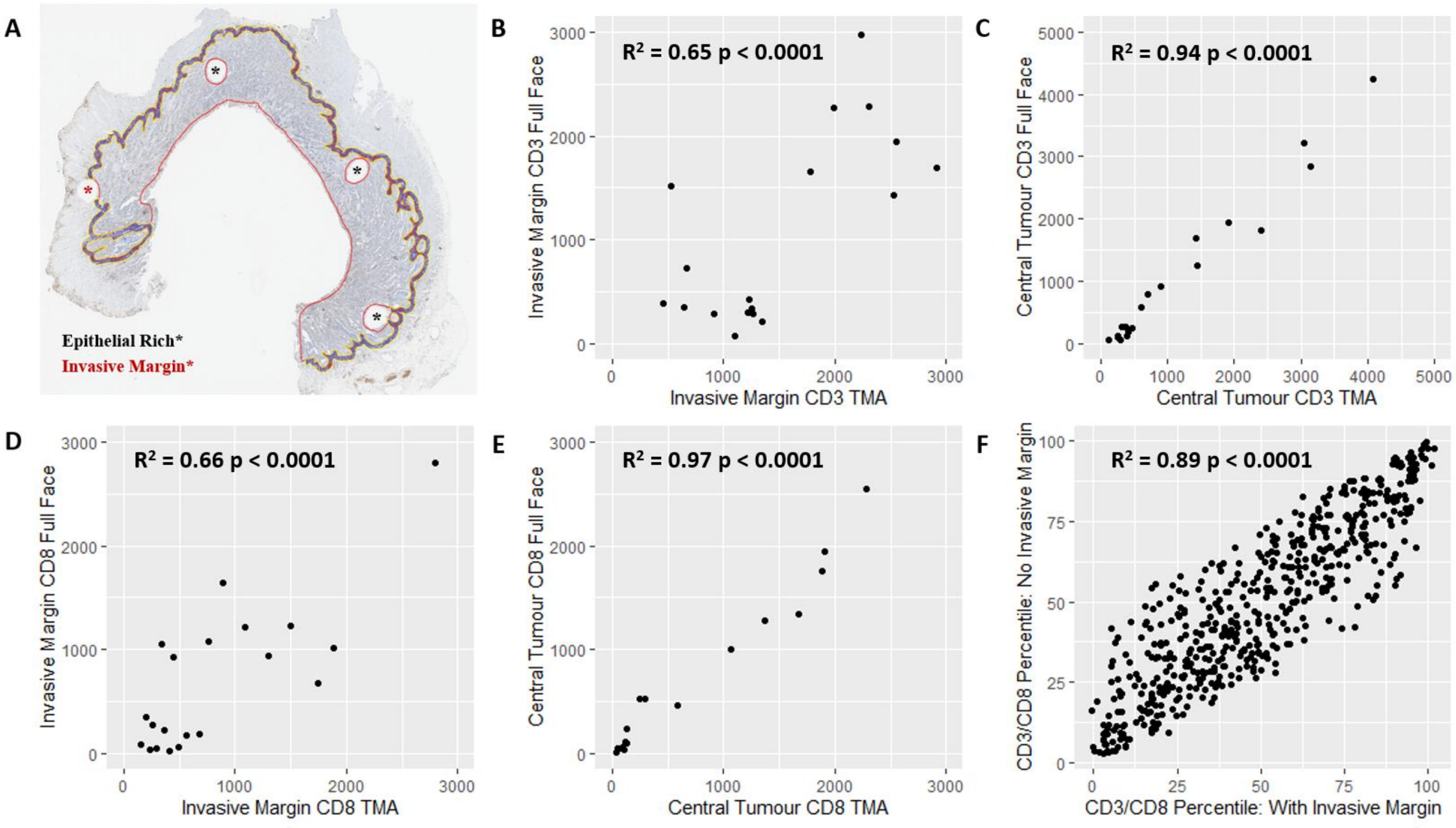

Supplementary Figure S2: Representative full-face tissue section of CRC stained for CD3 IHC with image analysis overlay demonstrating the annotations generated on full-face images and where the TMA cores were taken from (A). Plots demonstrating the monotonic relationship between CD3 and CD8 IHC expression on full-face sections and TMAs (B-E). Plot demonstrating the monotonic relationship between combined CD3 and CD8 IHC expression demonstrated as percentiles with the invasive margin and combined CD3 and CD8 IHC expression demonstrated as percentiles generated without the invasive margin (F).

Spearman's rank-order correlation was used to compare monotonic relationships between immune biomarkers in (B-F).

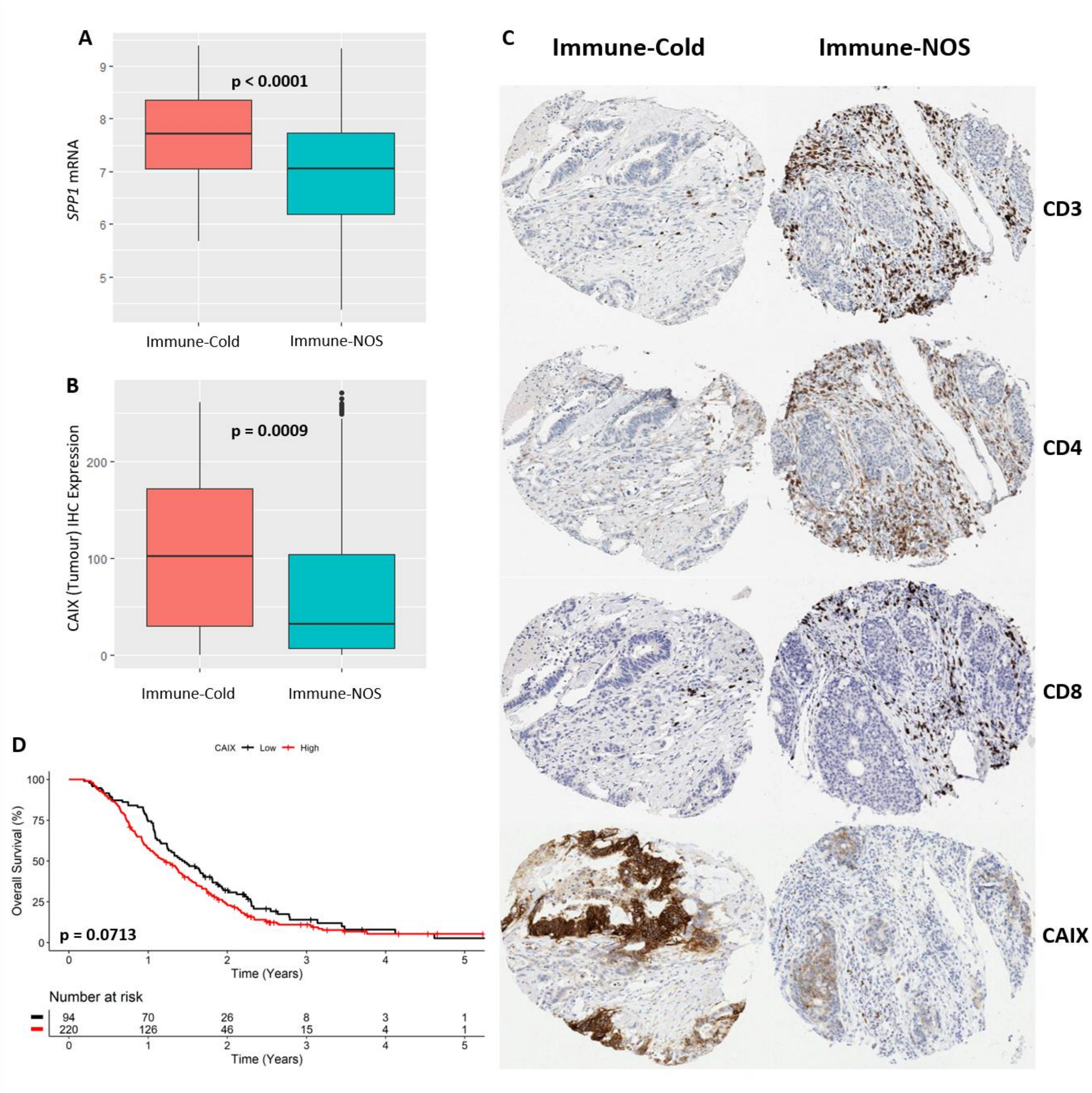

Supplementary Figure S3: Boxplots of *SPP1* mRNA (A) and CAIX IHC (B) expression by immune subgroups (immune-cold vs. immune NOS) in the S:CORT FOCUS cohort. Representative images of CD3, CD4, CD8, and CAIX IHC staining in immune subgroups (C). Kaplan-Meier curve of CAIX tumour IHC expression in the S:CORT FOCUS cohort (D).

Differences in immune subgroups were compared using ANOVA. Differences in survival curves are presented as Log-Rank p value. Immune-cold = patient stratification by collective low-density cell counts for CD3, CD4, and CD8 IHC; Immune-not otherwise specified (NOS) = any other combination of CD3, CD4, and CD8 IHC expression.

Biomarker	Antibody	Catalog Number	Clone	Source	Immunostainer	Antigen Retrieval	Dilution	Incubation	Detection Chemistry	Positive Control
CD3	CONFIRM anti-CD3	790-4341	2GV6	Ventana	Benchmark XT	CC1 for 32 minutes	Neat	16 minutes at 37oC	Optiview DAB	Tonsil
CD4	CONFIRM anti-CD4	790-4423	SP35	Ventana	Benchmark XT	CC1 for 60 minutes	Neat	16 minutes at 37oC	Ultraview DAB	Tonsil
CD8	Anti-CD8	M7103	CD/144B	Dako	Leica Bond RX	ER2 for 20 minutes	1:50	15 minutes at room temperature	Bond Polymer Refine Detection and Enhancer	Tonsil
CD20	Anti-CD20	M0755	L26	Dako	Leica Bond RX	ER1 for 30 minutes	1:400	15 minutes at room temperature	Bond Polymer Refine Detection and Enhancer	Tonsil
CAIX	Anti-CAIX	Ab15086	Poly.	Abcam	DAKO Autostainer Link 48	High pH Buffer	1:1500	20 minutes at room temperature	Envision FLEX+, HRP	Renal carcinoma
FOXP3	Anti-FOXP3	LS-C210349	SP97	LSBio	Benchmark XT	CC1 for 48 minutes	1:50	60 minutes at room temperature	Optiview DAB and Amplifier	Tonsil
ICOS	Anti-ICOS	#89601	D1K2T	Cell Signalling	Leica Bond RX	ER2 for 20 minutes	1:400	15 minutes at room temperature	Bond Polymer Refine Detection and Enhancer	Tonsil
IDO	Anti-IDO	#86630	D5J4E	Cell Signalling	Leica Bond RX	ER2 for 20 minutes	1:400	15 minutes at room temperature	Bond Polymer Refine Detection and Enhancer	Tonsil
PD-L1	VENTANA PD-L1	790-4905	SP263	Ventana	Benchmark XT	CC1 for 64 minutes	Neat	20 minutes at room temperature	Optiview DAB	Tonsil
CD3	Anti-CD3	790-4341	2GV6	Ventana	Leica Bond RX	ER1 for 30 minutes	1 in 6	30 minutes at room temperature	Opal Multiplex IHC	Tonsil
CD4	Anti-CD4	790-4423	SP35	Ventana	Leica Bond RX	ER1 for 30 minutes	1 in 4	30 minutes at room temperature	Opal Multiplex IHC	Tonsil
CD8	Anti-CD8	M7103	C8/144B	Dako	Leica Bond RX	ER1 for 30 minutes	1 in 400	30 minutes at room temperature	Opal Multiplex IHC	Tonsil

Supplementary Table S1: List of antibodies used in the study.

		Epi700 CRC															
		**Univariate**	**Multivariate***														
			**CD3**	**CD4**	**cCD4**	**CD8**	**CD20**	**FOXP3**	**ICOS**	**IDO-1: T**	**IDO-1: S**	**PD-L1: T**	**PD-L1: S**	**CD3+ CD8**	**CD3+ CD4+ CD8**	**CD3+ cCD4 +CD8**	**CD3+ CD4+CD8 +IDO-1:S**
Age																	
<70 : 70+	HR	1.81	1.37	1.35	1.35	1.36	1.33	1.34	1.36	1.39	1.40	1.39	1.36	1.36	1.35	1.34	1.38
	95% CI	(1.36-2.42)	(1.00-1.87)	(0.99-1.85)	(0.99-1.84)	(1.00-1.86)	(0.97-1.82)	(0.98-1.83)	(1.00-1.86)	(1.02-1.90)	(1.02-1.90)	(1.02-1.90)	(0.99-1.85)	(1.00-1.86)	(0.99-1.84)	(0.98-1.83)	(1.01-1.88)
	p value	<0.0001	0.0468	0.0556	0.0589	0.0489	0.0734	0.0645	0.0536	0.0378	0.0354	0.0388	0.0551	0.0491	0.0564	0.0625	0.0422
Sex																	
Male : Female	HR	0.99	0.94	0.97	0.94	0.94	0.95	0.97	1.01	0.98	0.98	0.98	0.97	0.95	0.98	0.97	1.00
	95% CI	(0.75-1.30)	(0.71-1.24)	(0.74-1.29)	(0.74-1.29)	(0.71-1.24)	(0.72-1.26)	(0.74-1.29)	(0.76-1.34)	(0.74-1.30)	(0.74-1.30)	(0.74-1.30)	(0.73-1.28)	(0.72-1.25)	(0.74-1.30)	(0.73-1.28)	(0.76-1.33)
	p value	0.9470	0.6510	0.8589	0.8574	0.6413	0.7424	0.8543	0.9237	0.9049	0.8921	0.9067	0.8074	0.7027	0.8956	0.8331	0.9799
MSI Status																	
Stable : High	HR	0.68	0.78	0.76	0.73	0.81	0.76	0.74	0.76	0.79	0.81	0.83	0.76	0.78	0.75	0.74	0.81
	95% CI	(0.47-0.98)	(0.54-1.15)	(0.52-1.11)	(0.50-1.07)	(0.55-1.19)	(0.52-1.12)	(0.50-1.08)	(0.52-1.11)	(0.54-1.17)	(0.55-1.19)	(0.56-1.22)	(0.52-1.12)	(0.53-1.14)	(0.51-1.10)	(0.51-1.09)	(0.55-1.18)
	p value	0.0382	0.2141	0.1508	0.1041	0.2877	0.1643	0.1146	0.1550	0.2376	0.2826	0.3403	0.1658	0.1941	0.1387	0.1269	0.2696
Stable : Missing	HR	0.94	1.04	1.09	0.96	1.04	1.02	1.02	1.05	1.01	0.99	1.06	1.04	1.05	1.07	0.99	1.04
	95% CI	(0.57-1.55)	(0.63-1.72)	(0.66-1.81)	(0.58-1.58)	(0.63-1.72)	(0.62-1.69)	(0.62-1.68)	(0.64-1.73)	(0.61-1.67)	(0.60-1.64)	(0.64-1.75)	(0.63-1.71)	(0.63-1.73)	(0.65-1.77)	(0.60-1.64)	(0.63-1.72)
	p value	0.7988	0.8801	0.7284	0.8599	0.8692	0.9254	0.9405	0.8536	0.9687	0.9748	0.8275	0.8878	0.8600	0.7824	0.9707	0.8697
TNM																	
Stage II : Stage III	HR	1.72	3.05	2.94	3.13	3.05	2.99	2.98	2.93	3.07	2.92	2.91	2.93	3.02	2.93	3.07	2.88
	95% CI	(1.31-2.26)	(2.25-4.13)	(2.17-3.97)	(2.31-4.24)	(2.25-4.13)	(2.21-4.05)	(2.20-4.04)	(2.16-3.98)	(2.26-4.15)	(2.16-3.95)	(2.15-3.94)	(2.16-3.97)	(2.23-4.08)	(2.16-3.96)	(2.26-4.14)	(2.13-3.90)
	p value	<0.0001	<0.0001	<0.0001	<0.0001	<0.0001	<0.0001	<0.0001	<0.0001	<0.0001	<0.0001	<0.0001	<0.0001	<0.0001	<0.0001	<0.0001	<0.0001
Chemotherapy																	
No : Yes	HR	0.49	0.29	0.30	0.27	0.28	0.29	0.29	0.30	0.28	0.30	0.29	0.29	0.29	0.30	0.28	0.30
	95% CI	(0.34-0.69)	(0.19-0.43)	(0.20-0.45)	(0.18-0.42)	(0.19-0.43)	(0.19-0.44)	(0.19-0.44)	(0.20-0.45)	(0.18-0.42)	(0.20-0.45)	(0.19-0.44)	(0.19-0.44)	(0.19-0.43)	(0.20-0.45)	(0.19-0.43)	(0.20-0.45)
	p value	<0.0001	<0.0001	<0.0001	<0.0001	<0.0001	<0.0001	<0.0001	<0.0001	<0.0001	<0.0001	<0.0001	<0.0001	<0.0001	<0.0001	<0.0001	<0.0001
CD3																	
High : Low	HR	1.41	1.45														
	95% CI	(1.04-1.92)	(1.06-1.99)	..	..	..	..	..	..	..	..	..	..	..	..	..	..
	p value	0.0280	0.0186														

CD4																	
	HR	1.80		1.68													
High : Low	95% CI	(1.36-2.38)	..	(1.27-2.23)	..	..	..	..	..	..	..	..	..	..	..	..	..
	p value	<0.0001		0.0003													
Calculated CD4																	
	HR	1.43			1.58												
High : Low	95% CI	(1.08-1.88)	..	..	(1.20-2.09)	..	..	..	..	..	..	..	..	..	..	..	..
	p value	0.0116			0.0012												
CD8																	
	HR	1.51				1.55											
High : Low	95% CI	(1.10-2.08)	..	..	..	(1.12-2.15)	..	..	..	..	..	..	..	..	..	..	..
	p value	0.0109				0.0077											
CD20																	
	HR	1.36					1.31										
High : Low	95% CI	(1.03-1.79)	..	..	..	..	(1.00-1.73)	..	..	..	..	..	..	..	..	..	..
	p value	0.0282					0.0529										
FOXP3																	
	HR	1.45						1.40									
High : Low	95% CI	(1.11-1.91)	..	..	..	..	..	(1.07-1.84)	..	..	..	..	..	..	..	..	..
	p value	0.0069						0.0156									
ICOS																	
	HR	1.66							1.54								
High : Low	95% CI	(1.26-2.18)	..	..	..	..	..	..	(1.17-2.05)	..	..	..	..	..	..	..	..
	p value	0.0003							0.0024								
IDO-1: T																	
	HR	1.34								1.49							
High : Low	95% CI	(1.02-1.75)	..	..	..	..	..	..	..	(1.12-1.97)	..	..	..	..	..	..	..
	p value	0.0376								0.0055							
IDO-1: S																	
	HR	1.97									1.91						
High : Low	95% CI	(1.31-2.96)	..	..	..	..	..	..	..	..	(1.26-2.88)	..	..	..	..	..	..
	p value	0.0010									0.0021						
PD-L1:T																	

	HR	1.52										1.47					
High : Low	95% CI	(1.12-2.06)	..	..	..	..	..	..	..	..	..	(1.07-2.02)	..	..	..	..	..
	p value	0.0071										0.0181					
PD-L1: S																	
	HR	1.33											1.22				
High : Low	95% CI	(1.01-1.75)	..	..	..	..	..	..	..	..	..	..	(0.92-1.62)	..	..	..	..
	p value	0.0405											0.1613				
CD3+CD8																	
	HR	1.34												1.35			
High : Low	95% CI	(1.00-1.79)	..	..	..	..	..	..	..	..	..	..	..	(1.01-1.81)	..	..	..
	p value	0.0475												0.0454			
CD3+CD4+CD8																	
	HR	1.85													1.70		
High : Low	95% CI	(1.39-2.45)	..	..	..	..	..	..	..	..	..	..	..	..	(1.28-2.27)	..	..
	p value	<0.0001													0.0003		
CD3+ Calculated CD4+CD8																	
	HR	1.33														1.39	
High : Low	95% CI	(1.01-1.74)	..	..	..	..	..	..	..	..	..	..	..	..	..	(1.06-1.83)	..
	p value	0.0402														0.0172	
CD3+CD4+CD8 +IDO-1:S																	
	HR	0.61															0.63
Mixed : High	95% CI	(0.41-0.93)	..	..	..	..	..	..	..	..	..	..	..	..	..	..	(0.41-0.97)
	p value	0.0223															0.0374
	HR	1.70															1.61
Mixed : Low	95% CI	(1.26-2.28)	..	..	..	..	..	..	..	..	..	..	..	..	..	..	(1.20-2.17)
	p value	0.0005															0.0017

Supplementary Table S2: Univariate and multivariable analysis for overall survival by all biomarkers in Epi700 study patients.

Data are hazard ratios (95% CI) and corresponding p values; Cox proportional-hazards were conducted based on reference category: target category for each covariate listed. IDO-1/PD-L1: T = IDO-1/PD-L1 tumour expression; IDO-1/PD-L1: S = IDO-1/PD-L1 stromal expression. *multivariable model mutually adjusted for Age, Sex, MSI Status, TNM Staging and Biomarker of Interest.

Epi700 CRC					Grampian CRC					S:CORT FOCUS				
Model	AICc	Delta AICc	AICcWt	LL	Model	AICc	Delta AICc	AICcWt	LL	Model	AICc	Delta AICc	AICcWt	LL
CD3+CD4+CD8	2547.951	1.0720	0.2581	-1272.972	CD3/CD4/CD8	2496.375	0.0000	0.6250	-1247.184	CD3/CD4/CD8	2717.400	0.0000	0.8937	-1357.694
CD3+CD8	2560.438	13.5589	0.0005	-1279.216	CD3/CD8	2499.897	3.5224	0.1074	-1248.945	CD3/CD8	2729.846	12.4459	0.0018	-1363.917
CD4+CD8	2546.879	0.0000	0.4411	-1272.436	CD4/CD8	2498.987	2.6123	0.1693	-1248.490	CD4/CD8	2722.705	5.3048	0.0630	-1360.346
CD3	2559.409	12.5293	0.0008	-1278.701	CD3	2500.271	3.8966	0.0891	-1249.132	CD3	2730.761	13.3611	0.0011	-1364.374
CD4	2548.338	1.4588	0.2127	-1273.166	CD4	2505.446	9.0709	0.0067	-1251.719	CD4	2723.616	6.2166	0.0399	-1360.802
CD8	2557.565	10.6861	0.0021	-1277.779	CD8	2507.393	11.0184	1.0000	-1252.693	CD8	2732.320	14.9198	0.0005	-1365.153
CD20	2559.619	12.7394	0.0008	-1278.806	..	..	..	..	..	..	..	..	..	..
FOXP3	2557.173	10.2936	0.0026	-1277.583	..	..	..	..	..	..	..	..	..	..
ICOS	2552.108	5.2289	0.0323	-1275.051	..	..	..	..	..	..	..	..	..	..
IDO-1: Tumour	2560.234	13.3543	0.0006	-1279.113	..	..	..	..	..	..	..	..	..	..
IDO-1: Stroma	2551.672	4.7922	0.0402	-1274.832	..	..	..	..	..	..	..	..	..	..
PD-L1: Tumour	2556.795	9.9154	0.0031	-1277.394	..	..	..	..	..	..	..	..	..	..
PD-L1: Stroma	2560.354	13.4752	0.0005	-1279.174	..	..	..	..	..	..	..	..	..	..
CD3+CD4+CD8+IDO1: Stroma	2556.479	9.5999	0.0036	-1277.236	..	..	..	..	..	..	..	..	..	..
Calculated CD4	2558.903	12.0232	0.0011	-1278.448	..	..	..	..	..	..	..	..	..	..
CD3+CalculatedCD4+CD8	2564.488	17.6082	0.0001	-1281.240	..	..	..	..	..	..	..	..	..	..

Supplementary Table S3: Competitive model selection table based off unadjusted AICc and LL for OS in the Epi700 CRC, Grampian CRC and S:CORT FOCUS cohorts.

Variable	S:CORT FOCUS Study Population				Full MRC Focus Trial Population*			
Treatment Strategy	A:FOLFIRI (MdG->Ir)	B:FOLFIRI (MdG->IrMdG)	B:FOLFOX (MdG->OxMdG)	C:FOLFOX (OxMdG)	A:FOLFIRI (MdG->Ir)	B:FOLFIRI (MdG->IrMdG)	B:FOLFOX (MdG->OxMdG)	C:FOLFOX (OxMdG)
Number of patients	156	66	83	80	710	356	356	357
Ratio of Study patients assigned to treatment	2	1	1	1	2	1	1	1
Age: median (years)	64	65	64	62	63	64	64	64
Age: interquartile range (years)	59-70	59-71	60-70	56-68	59-69	57-70	50-69	54-69
Colon as primary site (Percentage)	90	94	96	95	69	65	67	66
Median Overall Survival (months)	13.8	16.4	16.0	14.9	13.9	15	15.2	15.4

Supplementary Table S4: Comparability of chemotherapy cohort with the Full MRC FOCUS trial population*.

*Excluding patients who received first-line FOLFIRI.

		Epi700 CRC			Grampian CRC			S:CORT FOCUS			Pooled CRC*		
		Univariate	Multivariable (CD3+CD8)	Multivariable (CD3+CD4+CD8)	Univariate	Multivariable (CD3+CD8)	Multivariable (CD3+CD4+CD8)	Univariate	Multivariable (CD3+CD8)	Multivariable (CD3+CD4+CD8)	Univariate	Multivariable (CD3+CD8)	Multivariable (CD3+CD4+CD8)
Age													
	HR	1.81	1.34	1.37	1.49	1.64	1.65	1.10	1.12	1.15	1.42	1.30	1.33
<70 : 70+	95% CI	(1.36-2.42)	(0.99-1.83)	(1.00-1.86)	(1.12-1.98)	(1.23-2.18)	(1.24-2.19)	(0.83-1.44)	(0.85-1.48)	(0.88-1.52)	(1.21-1.66)	(1.11-1.53)	(1.13-1.56)
	p value	<0.0001	0.0622	0.0493	0.0058	0.0007	0.0006	0.5050	0.4177	0.3050	<0.0001	0.0015	0.0006
Sex													
	HR	0.99	0.99	0.99	1.17	1.03	1.03	1.13	1.14	1.10	1.10	1.06	1.05
Male : Female	95% CI	(0.75-1.30)	(0.75-1.32)	(0.75-1.31)	(0.89-1.54)	(0.79-1.36)	(0.78-1.36)	(0.88-1.45)	(0.89-1.47)	(0.86-1.42)	(0.94-1.28)	(0.91-1.24)	(0.90-1.22)
	p value	0.9470	0.9541	0.9311	0.2520	0.8206	0.8233	0.3230	0.2949	0.4330	0.2270	0.4273	0.5364
MSI Status													
	HR	0.68	0.74	0.73	1.14	1.10	1.08	4.10	4.54	4.92	0.99	1.04	1.03
Stable : High	95% CI	(0.47-0.98)	(0.50-1.08)	(0.50-1.07)	(0.80-1.62)	(0.77-1.57)	(0.76-1.56)	(2.27-7.42)	(2.49-8.30)	(2.69-9.00)	(0.78-1.26)	(0.81-1.32)	(0.81-1.31)
	p value	0.0382	0.1162	0.1038	0.4787	0.6073	0.6624	<0.0001	<0.0001	<0.0001	0.9550	0.7796	0.8322
	HR	0.94	1.02	1.03	2.92	2.24	2.08	0.87	0.88	0.89	0.99	1.01	1.01
Stable : Missing	95% CI	(0.57-1.55)	(0.62-1.69)	(0.63-1.70)	(1.29-6.60)	(0.98-5.11)	(0.91-4.75)	(0.58-1.30)	(0.58-1.32)	(0.59-1.33)	(0.74-1.33)	(0.75-1.36)	(0.75-1.36)
	p value	0.7988	0.9236	0.9024	0.0101	0.5423	0.0834	0.4890	0.5326	0.5570	0.9560	0.9584	0.9433
TNM													
	HR	1.72	2.98	2.98	3.13	3.06	3.10				2.27	3.06	3.05
Stage II : Stage III	95% CI	(1.31-2.26)	(2.21-4.04)	(2.20-4.03)	(2.31-4.23)	(2.25-4.17)	(2.28-4.21)	..	..	..	(1.86-2.76)	(2.48-3.79)	(2.47-3.78)
	p value	<0.0001	<0.0001	<0.0001	<0.0001	<0.0001	<0.0001				<0.0001	<0.0001	<0.0001
Chemotherapy													
	HR	0.49	0.29	0.29							0.48	0.28	0.28
No : Yes	95% CI	(0.34-0.69)	(0.19-0.44)	(0.19-0.44)	..	..	..	..	..	..	(0.34-0.69)	(0.19-0.40)	(0.19-0.40)
	p value	<0.0001	<0.0001	<0.0001							<0.0001	<0.0001	<0.0001
CD3+CD8													
	HR	1.46	1.37		1.97	1.71		1.23	1.31		1.48	1.41	
Immune-NOS : Immune-Cold	95% CI	(1.07-2.01)	(0.99-1.90)	..	(1.48-2.64)	(1.28-2.30)	..	(0.97-1.57)	(1.02-1.67)	..	(1.26-1.74)	(1.20-1.66)	..
	p value	0.0189	0.0541		<0.0001	0.0003		0.0869	0.0315		<0.0001	<0.0001	
CD3+CD4+CD8													
	HR	1.64		1.60	2.16		1.94	1.86		1.98	1.90		1.84
Immune-NOS : Immune-Cold	95% CI	(1.15-2.36)	..	(1.11-2.30)	(1.61-2.91)	..	(1.43-2.62)	(1.39-2.50)	..	(1.47-2.67)	(1.59-2.28)	..	(1.54-2.21)
	p value	0.0065		0.0110	<0.0001		<0.0001	<0.0001		<0.0001	<0.0001		<0.0001

Supplementary Table S5: Univariate and multivariable analysis for immune subgroups (defined by either CD3 and CD8 IHC or CD3, CD4 and CD8 IHC) for overall survival in study patients.

Data are hazard ratios (95%); Cox proportional-hazards were conducted based on reference category: target category for each covariate listed. Immune-not otherwise specified (NOS) = any other density of immune cell combinations; Immune-cold = combined low density of immune cells assessed. *Observations stratified by cohort.

Column ID	Gene Symbol	p Value (A vs. B)	Fold Change (A vs. B)	Fold Change (A vs. B)
ADXECEMUTR.7326_x_at	PLIN2	7.30E-07	2.14589	A up vs B
ADXECEMUTR.7326_at	PLIN2	2.73E-07	2.1087	A up vs B
ADXEC.2281.C1_x_at	SPP1	6.85E-06	1.81233	A up vs B
ADXEC.18903.C1_at	---	5.82E-06	1.73532	A up vs B
ADXECAD.20996_s_at	SERPINE1	2.63E-06	1.6871	A up vs B
ADXOCEC.14560.C1_at	SPP1	4.25E-06	1.65194	A up vs B
ADXEC.2870.C2_s_at	ADM	3.94E-06	1.63129	A up vs B
ADXECAD.5047_at	SPP1	5.61E-06	1.62136	A up vs B
ADXEC.2281.C2_x_at	SPP1	6.01E-06	1.61439	A up vs B
ADXEC.8484.C1_s_at	SERPINE1	9.11E-06	1.5872	A up vs B
ADXEC.20355.C2_s_at	TRBC1 /// TRBV19	9.55E-06	-1.51032	A down vs B
ADXEC.20355.C1_x_at	TRBC2 /// TRBV3-1 /// TRBV5-4 /// TRBV6-5	9.29E-06	-1.52312	A down vs B
ADXEC.4367.C1_at	CCL11	8.17E-06	-1.64526	A down vs B
ADXEC.20355.C1_s_at	TRBC2 /// TRBV3-1 /// TRBV5-4 /// TRBV6-5 /// TRBV7-2	1.37E-06	-1.70235	A down vs B
ADXEC.4367.C1_x_at	CCL11	7.08E-06	-1.7116	A down vs B
ADXECNTDJ.2384_s_at	IGHA1	7.33E-06	-1.77158	A down vs B
ADXEC.7864.C1-a_s_at	FOXF1	6.51E-06	-2.03022	A down vs B
ADXECNTDJ.3332_x_at	CXCL14	1.31E-05	-2.05025	A down vs B
ADXEC.111.C193_s_at	IGH /// IGHA1 /// IGHA2	2.80E-07	-2.22843	A down vs B
ADXEC.9374.C1_at	CXCL14	1.11E-05	-2.43771	A down vs B

Supplementary Table S6: List of 20 differentially expressed probes between immune subgroups A and B* in S:CORT FOCUS cohort transcriptional profiles.

*Group A (Immune-cold) = patient stratification by collective low-density cell counts for CD3, CD4, and CD8 IHC; Group B (Immune-not otherwise specified) = any other combination of CD3, CD4, and CD8 IHC expression.

Gene Symbol	Number of Occurrences
SPP1	4
PLIN2	2
SERPINE1	2
CCL11	2
CXCL14	2
IGH /// IGHA1 /// IGHA2	1
TRBC2 /// TRBV3-1 /// TRBV5-4 /// TRBV6-5 /// TRBV7-2	1
ADM	1
---	1
FOXF1	1
IGHA1	1
TRBC2 /// TRBV3-1 /// TRBV5-4 /// TRBV6-5	1
TRBC1 /// TRBV19	1

Supplementary Table S7: List of top gene occurrences in the 20 most differentially expressed probes list shown in Supplementary Table S6.

	Univariate	p value	Multivariable	p value
Age				
<70 : 70+	1.04 (0.78-1.38)	0.7840	1.10 (0.83-1.47)	0.5051
Sex				
Male : Female	1.08 (0.84-1.40)	0.5420	1.07 (0.83-1.38)	0.6181
MSI Status				
Stable : High	4.28 (2.31-7.94)	<0.0001	4.73 (2.48-9.01)	<0.0001
Chemotherapy				
5FU : FOLFOX	1.09 (0.85-1.40)	0.4840	1.22 (0.94-1.59)	0.1395
CRIS				
CRIS A : CRIS B	1.53 (1.01-2.32)	0.0448	1.61 (1.06-2.46)	0.0251
CRIS A : CRIS C	0.67 (0.46-0.98)	0.0375	0.79 (0.53-1.19)	0.2582
CRIS A : CRIS D	0.53 (0.32-0.88)	0.0143	0.74 (0.43-1.26)	0.2660
CRIS A : CRIS E	0.69 (0.45-1.06)	0.0933	0.84 (0.54-1.31)	0.4423
CRIS A : Unclassified	0.69 (0.45-1.06)	0.0885	0.92 (0.57-1.47)	0.7208
KRAS				
Low : High	1.13 (0.89-1.45)	0.3280	0.94 (0.72-1.24)	0.6832
Immune Group				
Immune-NOS + Hypoxia-Low : Immune-NOS + Hypoxia-High	1.54 (1.16-2.04)	0.0025	1.55 (1.12-2.15)	0.0078
Immune-NOS + Hypoxia-Low : Immune-Cold	2.20 (1.55-3.11)	<0.0001	2.19 (1.48-3.25)	<0.0001

Supplementary Table S8: Univariate and multivariable analysis for immune subgroups defined by the density of CD3, CD4 and CD8 expressing cells and tumour hypoxia for overall survival in S:CORT FOCUS patients.

Data are hazard ratios (95%); Cox proportional-hazards were conducted based on reference category: target category for each covariate listed. Immune-not otherwise specified (NOS) + Hypoxia-Low = any other density of CD3, CD4 and CD8 IHC expression and low tumour hypoxia based on the Winters Hypoxia Metagene signature; Immune-not otherwise specified (NOS) + Hypoxia-High = any other density of CD3, CD4 and CD8 IHC expression and high tumour hypoxia based on the Winters Hypoxia Metagene signature; Immune-cold = low-density cell counts for CD3, CD4 and CD8 IHC expression only.
